# Emotion Facial Processing in Children With Autism Spectrum Disorder: A Pilot Study of the Impact of Service Dogs

**DOI:** 10.3389/fpsyg.2022.869452

**Published:** 2022-05-20

**Authors:** Nicolas Dollion, Marine Grandgeorge, Dave Saint-Amour, Anthony Hosein Poitras Loewen, Nathe François, Nathalie M. G. Fontaine, Noël Champagne, Pierrich Plusquellec

**Affiliations:** ^1^Univ Rennes, Normandie Univ., CNRS, EthoS (Éthologie Animale et Humaine) – UMR 6552, Rennes, France; ^2^Laboratoire d’Observation et d’Éthologie Humaine du Québec, Montréal Mental Health University Institute, Centre Intégré Universitaire de Santé et de Services Sociaux de l’Est-de-l’Île-de-Montréal (CIUSSS Est), Montréal, QC, Canada; ^3^School of Psychoeducation, University of Montreal, Montréal, QC, Canada; ^4^Mira Foundation Inc., Sainte-Madeleine, QC, Canada; ^5^Department of Psychology, Centre de Recherche en Neuroscience Cognitives, NeuroQAM, Université du Quebec à Montréal, Montréal, QC, Canada; ^6^School of Criminology, Université de Montréal, Montréal, QC, Canada; ^7^Centre Interdisciplinaire de Recherche sur le Cerveau et l’Apprentissage, University of Montréal, Montréal, QC, Canada

**Keywords:** autism spectrum disorder (ASD), facial expression processing, eye-tracking, service dog, emotion recognition

## Abstract

Processing and recognizing facial expressions are key factors in human social interaction. Past research suggests that individuals with autism spectrum disorder (ASD) present difficulties to decode facial expressions. Those difficulties are notably attributed to altered strategies in the visual scanning of expressive faces. Numerous studies have demonstrated the multiple benefits of exposure to pet dogs and service dogs on the interaction skills and psychosocial development of children with ASD. However, no study has investigated if those benefits also extend to the processing of facial expressions. The aim of this study was to investigate if having a service dog had an influence on facial expression processing skills of children with ASD. Two groups of 15 children with ASD, with and without a service dog, were compared using a facial expression recognition computer task while their ocular movements were measured using an eye-tracker. While the two groups did not differ in their accuracy and reaction time, results highlighted that children with ASD owning a service dog directed less attention toward areas that were not relevant to facial expression processing. They also displayed a more differentiated scanning of relevant facial features according to the displayed emotion (i.e., they spent more time on the mouth for joy than for anger, and vice versa for the eyes area). Results from the present study suggest that having a service dog and interacting with it on a daily basis may promote the development of specific visual exploration strategies for the processing of human faces.

## Introduction

Autism spectrum disorder (ASD) is a complex and heterogeneous condition, whose symptoms persist over time and development. Apart from repetitive and restricted interests and behaviors (including stereotypic behaviors), this disorder is also characterized by social interaction difficulties as well as by communication impairments (both verbal and non-verbal) (DSM-5; American Psychiatric Association, 2013), with implications on several daily life skills.

Being able to decode and understand others’ facial expressions is a skill that plays a pivotal role in non-verbal communication and social interaction. This capacity allows one to infer emotional states and to access, at least partially, others’ intentions, which fosters the establishment and maintenance of social interaction. It thus promotes behavioral adaptation and regulation of interaction strategies, and is involved in the Theory of Mind (i.e., the capacity to attribute mental states to others) and in the development of empathetic feelings (Darwin, 1872; Izard et al., 2001). Numerous studies have showed that individuals with ASD have lifespan difficulties with recognizing and understanding others’ facial expressions (*Review*: Jemel et al., 2006; Harms et al., 2010; Lozier et al., 2014; *Children*: Gross, 2004; Nagy et al., 2021; *Adolescents*: Smith et al., 2010; Wingenbach et al., 2017; *Adults*: Pelphrey et al., 2002; Clark et al., 2008), with poorer accuracy and increased response time in individuals with ASD compared to neurotypicals (NT) (Celani et al., 1999; Smith et al., 2010; Wingenbach et al., 2017; Loth et al., 2018; Nagy et al., 2021). Although these issues in emotion processing seems to be general, different studies highlight that the recognition of negative facial expressions could be particularly affected (i.e., anger, disgust, fear, sadness, and negative surprise) (Pelphrey et al., 2002; Smith et al., 2010; Lozier et al., 2014; Evers et al., 2015; Wingenbach et al., 2017). In addition, in their meta-analysis, Lozier et al. (2014) revealed that these difficulties in facial expression recognition in ASD were not only present across development, but that the magnitude of differences compared to NT increased with age (i.e., recognition abilities remain essentially flat over time in individuals with ASD while it is steadily improving across development in NT). Also of note, the magnitude of facial expression recognition difficulties positively correlates with ASD symptoms severity (Humphreys et al., 2007; Evers et al., 2015; Loth et al., 2018) and children’s age (Smith et al., 2010; Lozier et al., 2014; Nagy et al., 2021).

Deficits in social attention and orientation toward faces in individuals with ASD are commonly invoked as a potential source of these difficulties in facial expression processing. A reduced attention to faces in individuals with ASD is often reported, along with a stronger exploration of other items from the visual scene (e.g., objects, bodies, etc.) (Klin et al., 2002, 2003; Riby and Hancock, 2008; Howard et al., 2019; Vacas et al., 2021). Alterations of visual exploration strategies of faces in individuals with ASD are also observed. Indeed, individuals with ASD explore less the inner features of faces relative to the rest of the face and/or of the visual scene (Pelphrey et al., 2002; Boraston and Blakemore, 2007; Speer et al., 2007; de Wit et al., 2008; Howard et al., 2019). Moreover, when exploring those features, they gaze less at the eyes compared to NT (Speer et al., 2007; Corden et al., 2008; de Wit et al., 2008; Kliemann et al., 2010; Black et al., 2017), but rather explore and rely more on the mouth area (Joseph and Tanaka, 2003; Gross, 2004; Dalton et al., 2005; Jemel et al., 2006; Spezio et al., 2007; Rutherford and Towns, 2008; Bird et al., 2011). Studies using eye-tracking demonstrate that the time individuals spent exploring the eye area is associated with a more efficient recognition of facial expression (e.g., Royer et al., 2018), which is also the case of children with ASD (Corden et al., 2008; Bal et al., 2010; Kliemann et al., 2010, 2012). Using the “Reading the Mind in the Eyes Test,” a task requesting participants to recognize emotions based on photographs depicting only the eye area of expressive faces, Baron-Cohen et al. (1997) identified in high functioning individuals with ASD the presence of a deficit in the Theory of Mind, i.e., the ability to infer mental states of others when only the eye area is available.

Expressing facial emotions requires the recruitment and differential activation of specific facial muscles (Ekman and Friesen, 1978). As such, efficient distinction and recognition of facial expressions involve the visual exploration of distinctive areas, which translates into a differential contribution of facial features (e.g., eyes, mouth, and eye-brows), and different visual exploration patterns according to the displayed emotion (Calder et al., 2000; Calvo and Nummenmaa, 2008; Calvo et al., 2018; Beaudry et al., 2014; Wells et al., 2016; Blais et al., 2017). Studies on NT individuals notably highlight that the eye area is more explored and contributes more to the recognition of anger, fear, and sadness, whereas the mouth area is more explored for joy (Smith et al., 2005; Eisenbarth and Alpers, 2011; Schurgin et al., 2014; Calvo et al., 2018). However, it has been shown that, in addition to atypical scanning strategies, this differentiation in visual exploration with respect to the displayed emotion is more limited in individuals with ASD (de Wit et al., 2008; Åsberg et al., 2017). Exploration strategies of faces and facial expressions are thus altered in ASD, which could be also at the root of the difficulties in facial expression recognition observed in ASD (Black et al., 2017), as they would result in an altered reading/decoding of facial expressions.

The alterations in face and facial expression exploration strategies are not homogeneous across individuals with ASD. As for social skills, several studies reported differences according to the symptoms and severity of ASD (Speer et al., 2007; Müller et al., 2016; Åsberg et al., 2017), as well as to the chronological age (Black et al., 2017). But another factor may play a greater role than expected up to now: experience with animals and especially with dogs. Research has shown multiple benefits of exposure to pet dogs and service dogs^1^ on the socio-emotional development of children with ASD (Viau et al., 2010; Carlisle, 2012, 2015; Berry et al., 2013; Grandgeorge, 2015; Wright et al., 2016; Carlisle et al., 2018, 2020). These benefits were observed through exposure to a dog during animal assisted intervention (Martin and Farnum, 2002; Silva et al., 2011, 2020; Funahashi et al., 2014; Ávila-Álvarez et al., 2020), as well as through the daily presence of a dog within the child’s life, either as a pet (Carlisle, 2012, 2015; Grandgeorge et al., 2012; Wright et al., 2015, 2016; Hall et al., 2016; Carlisle et al., 2020) or as a service dog (Hoffman, 2011; Brown, 2017; Sprod and Norwood, 2017; Bibbo et al., 2019; Leung et al., 2021). These studies notably depicted various benefits on interaction skills and psychosocial development of children with ASD, including improvement of social, language and communication skills, increase in prosocial behaviors and social interactions, and improvement in social reciprocity and empathetic feelings. From a theoretical point of view, these benefits rely on the specificities of the interaction with an animal. Compared to humans, animals, and especially dogs, rely more on non-verbal communication and their actions seem easier to decode and to be more predictable than human actions for children with ASD (Redefer and Goodman, 1989; Leslie, 1994; Prothmann et al., 2009; Grandgeorge and Hausberger, 2011; Grandgeorge et al., 2020). Furthermore, different studies showed that, contrarily to human faces, the processing of animal faces is not altered in individuals with ASD (i.e., they tend to process animal faces as well as NT individuals do). Indeed, in adolescents with ASD, the hypoactivation of face processing regions observed for human faces is not observed for animal faces (Whyte et al., 2016). Eye-tracking studies also showed that, contrarily to human faces, children with ASD show greater attention to animal faces (Dollion et al., 2021) and spend more time gazing at the eyes of animal faces compared to other facial areas (Muszkat et al., 2015; Grandgeorge et al., 2016; Valiyamattam et al., 2020). Additionally, Davidson et al. (2019) have recently shown that while children with ASD are less accurate in recognizing emotions on human faces, they are more accurate in recognizing canine emotions (i.e., anger, sadness, fear, joy, and neutral) and did not differ from NT children for emotions displayed by dog faces. Similar results have also been reported on adolescents with ASD exposed to expressive faces with an animal filter applied [i.e., item corresponding to pictures of inner features of a human face displaying emotions added on top of pictures of animal facial contours (lion or gorilla)] (Cross et al., 2019).

Taking these results together, one may ask if the impacts of animals on the communication and interaction skills of children with ASD also extend to the processing of facial expressions. The aim of the present study was thus to investigate if a service dog within the daily life of children with ASD could influence their facial recognition skills. To do so, 15 children with ASD with a service dog and 15 without were engaged in a computerized facial expression recognition task during which their accuracy and visual exploration patterns were measured using an eye tracker. We predicted that children with ASD living with a service dogs, compared to the ones without, would have greater facial expression processing skills, which would translate into (1) better accuracy and reaction time, (2) better general face scanning strategies (i.e., more attention toward inner facial features), and (3) more differentiated scanning strategies according to facial expressions (i.e., difference in the latency to gaze at and/or in time spent visually exploring the distinctive facial features according to the displayed expression).

## Materials and Methods

The present research was non-invasive and did not involve pharmacological intervention. All experiments were performed in accordance with the Declaration of Helsinki (6th revision). The procedure and protocol were approved by the University of Montreal’s Research Ethics committee in Education and Psychology (CERAS-2018 19-11-D). Informed consent was obtained from all individual participants included in the study. All parents provided written consent for their child’s involvement in this study and all children with ASD provided verbal and/or written approval for their participation.

### Participants

Fifteen children with ASD with a service a dog (14 boys and 1 girl; mean age, 166.7 ± 28.2 months) and 15 children with ASD without a service dog (9 boys and 6 girls; mean age, 141.4 ± 33.7 months) participated in this study (Table 1). Concerning children’s ethno-cultural group, among the 30 children with ASD who participated in this study, 27 were Caucasian and 3 were African (i.e., 2 in the group with a service dog and 1 the group without a service dog). All service dogs were trained and provided by the Mira Foundation (www.mira.ca), located in Quebec (Canada). The Mira Foundation is a non-profit organization which trains and donates service dog’s to individuals with visual and physical disabilities, as well as children with ASD and their families. The children had their service dog for at least 2.5 years (mean delay between dog placement and time of experimentation, 51.9 ± 13.4 months). Both groups did not differ for chronological age (Independent Student’s T-test, *T* = 1.62, *p* = 0.12). Children were recruited among the database of beneficiaries list (i.e., children with ASD already matched with a service dog for at least 2.5 years) and the waiting list (i.e., children already selected to receive a service dog based on their attraction toward dogs and waiting to be matched) of the Mira Foundation. The inclusion criteria were as follows: the child needed (1) to have a diagnosis of ASD delivered by a clinician (i.e., pediatrician and child–psychiatrist), (2) not to have a diagnosis of or suspicion of epilepsy, and (3) not to be wearing glasses with a strong correction [i.e., glasses that the child could not remove without a feeling of discomfort or causing clear vision difficulties (blurry vision, issues with details within a few meters, etc.)]. Additional criteria for the service dog group were that children needed to be aged between 5 and 15 years old by the time of service dog placement and not to have been the recipient of a service dog prior to this placement. Children in the group without a service dog (i.e., children from the waitlist – already selected to have a service dog) needed not to own a pet dog and not to have been the previous recipient of a service dog. For ethical reasons, children in the group without a service group were recruited, according to inclusion criteria, within the Mira Foundation’s waitlist among the selected candidates that were about to receive their service dog within the next few months.

**TABLE 1:** General characteristics of the children with ASD (with and without a service dog) included in the study.

Children with a service dog					Children without a service dog			
Subject number	Sex	Child’s age at observation (in years)	Child’s age at dog placement (in years)	Comorbidities	Subject number	Sex	Child’s age at observation (in years)	Comorbidities
1	M	11.37	7.02	ADHD	16	M	13.89	–
2	M	16.09	12.71	ADD	17	M	11.51	MD
3	M	12.48	9.77	ADHD–LDD	18	F	10.69	*
4	M	15.39	9.70	ADD–LDD–SPD	19	M	13.99	OD–AD–ADHD
5	F	11.46	6.55	–	20	M	12.45	ADHD–AD–LDD–LD–LeD
6	M	15.46	11.63	ADHD*	21	F	9.47	–
7	M	14.20	10.04	ADHD*	22	F	8.77	LD
8	M	19.49	13.14	ID	23	F	11.81	ADHD–AD–OD
9	M	11.18	8.04	ADHD–LDD	24	M	16.82	–
10	M	12.99	7.19	–	25	M	10.36	ADHD–VD–ED
11	M	17.56	13.09	ADD–MD	26	M	10.07	ADHD
12	M	12.36	7.47	ADHD	27	M	11.15	AD–SPD
13	M	12.67	9.28	ADHD–AD–MD	28	F	11.59	ADHD–AD
14	M	12.36	6.28	ADHD	29	M	12.82	–
15	M	14.18	10.77	MD–LDD	30	F	19.96	ADHD–T

*Comorbidity: AD, anxiety disorder; ADD, attentional deficit disorder; ADHD, attentional deficit with hyperactivity disorder; ED, eating disorder; ID, intellectual delay; LD, learning delay; LDD, language development disorder; LeD, learning disorder; MD, motor dyspraxia; OD, oppositional disorder; T, trichotillomania; SPD, sensory processing disorder; VD, verbal dyspraxia. Asterisk indicates children with an Asperger diagnostic.*

Autism spectrum disorder diagnostic was confirmed based on consultation of the full medical record transmitted by families to the Mira Foundation. Presence of other diagnostics (i.e., comorbidities) was also checked based on consultation of this record. Most of the participants (80%) had co-morbidities (e.g., attention deficit hyperactivity disorder, anxiety disorder, developmental delay; refer to Table 1 for details) and all were fully verbal (i.e., able to use full sentences to communicate). Nine additional children participated in the study but were not included in the final sample for analyses: five due to calibration failure, two due to intellectual deficiency compromising their understanding of the procedure, one due to a nystagmus, and one preferred to stop halfway through the experiment.

### Stimuli

Forty videos of expressive faces were used, with each video showing a single dynamic 3D avatar’s face displaying an emotion. Those expressive faces consisted in eight different avatars, four males and four females from two different ethnocultural groups (i.e., Caucasian and African) and two ages (i.e., teenager and adult), displaying five different emotions (i.e., anger, fear, joy, sadness, and neutral). Each video lasted 10.5 s and started with the avatar posing a neutral facial expression. When expressive, the avatar progressively changed reaching an expressivity peak (apex) at 10.5 s (i.e., morphing technique), whereas the avatar remained inexpressive during whole 10.5 s for the neutral expression. Creation of those stimuli was performed using an approach similar to Cigna et al. (2015). The avatars’ faces were generated using FaceGen Artist Pro (Singular Inversions Inc., Toronto, ON, Canada) and Daz Studio software (Daz Productions Inc., Salt Lake City, UT, United States), and their animation and shading were performed using Autodesk Mudbox (Autodesk Inc., San Rafael, CA, United States) and Unity Software (Unity Technologies Inc., San Francisco, CA, United States). Facial expressions were generated by applying the description of facial muscles activation and movement involved in each facial expression of emotions (Ekman and Friesen, 1971, 1978; Du and Martinez, 2015). The videos were 1,280 pixels width and 720 pixels height, and were mounted on a shaded gray background (example of a video available in Supplementary File 1).

### Apparatus and Procedure

Stimuli were presented on a 1,920 × 1,080 screen using Maltlab software (Mathworks, Natick, MA, United States) and the Psychtoolbox extension. During the experiment, children’s eye-movements were recorded using the TrackPixx Mini eye-tracking system (VPixx Technologies, Saint-Bruno, QC, Canada) with a sampling rate set at 120 Hz. Eye-movement data were recorded and extracted on both eyes using Matlab and Psychtoolbox, and were analyzed offline.

All experiments were performed in a dedicated room at the Mira Foundation. Upon arrival, a description of the study was given to the parents and their child, and informed written consent was obtained. Children were installed on a seat at an approximate distance of 60 cm in front of the screen. The eye movement-tracking system’s position as well as the child’s position were adjusted to optimize gaze detection before the experiment. Parents were present in the room, sitting on a chair at a small distance behind their child and were asked to remain silent during the experiment.

During the experiment, the 40 dynamic avatar videos were presented to the child. The experiment consisted of 4 blocks of 10 trials each, allowing the child to take a break between blocks if needed. The videos were randomly presented across all trials. Before each block a 16-point calibration phase was performed [i.e., sequential presentation of 16 targets (i.e., small white circle with a central red dot) on various locations on the screen that the participant had to fixate] in order to ensure correct gaze detection. The calibration procedure could be repeated if the precision of gaze detection was not acceptable. Each trial started with a fixation cross presented for 750 ms, followed by the video of one of the expressive avatars (10.5 s), then an answer screen was displayed until participant responded triggering a new trial. During the videos, children could indicate at any time when they recognized the displayed emotion by pressing on the mouse buttons. At their click, an answer screen, consisting of five pictograms (from the IdeoPicto emotion management set; IdeoPicto Inc., L’Assomption, QC, Canada) displaying the five possible emotions (anger, fear, joy, sadness, or neutral), appeared and the child had to click on the pictogram depicting the emotion they thought was displayed by the avatar. No time constrain was applied for the child to answer once the screen was displayed. If at the end of the video (i.e., 10.5 s) the child did not emit any click, the answer screen would automatically appear. This sequence was repeated for each trial. Children were instructed to try to be as fast and as accurate as they could.

### Data Analyses

**FIGURE 1:**
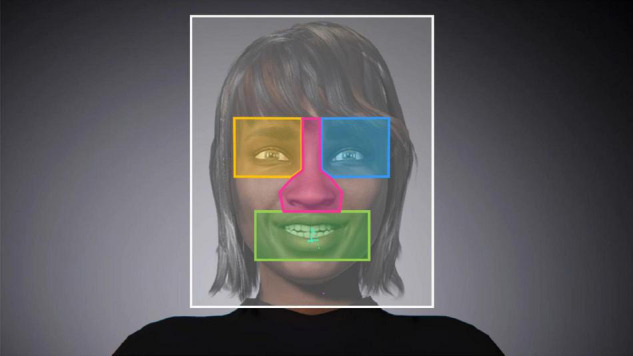
Illustration of the areas of interest (AOI) applied on the avatars’ faces for the extraction of oculometry data. *Left eye area* in yellow, *right eye area* in blue, *nose area* in pink, *mouth area* in green, *face contours area* in white, and *outside area* in gray.

Six areas of interest (AOI) were defined on the avatars: left eye, right eye, nose, mouth, face contours, and outside of the face (i.e., the rest of the visual scene). AOIs were build using rectangles and polygons (see Figure 1). Since avatars were dynamic, AOIs were defined to include the facial features while they moved within the target facial area across emotions for each avatar (e.g., eyebrows raising for the eye area). Landmarks were established and applied for drawing the AOIs in order to guarantee that they included the same features and were homogeneous across faces. Size of the AOIs could slightly vary between avatars’ faces, due to inter-avatar variability in the face’s proportions and traits. Attention was paid so that their proportion remained similar across avatars. Based on oculometric data, two variables were extracted: the sample duration (total time gaze was detected within an AOI, including fixation and saccade) and the latency of first sample (delay before first gaze detection within an AOI). The sample duration and the latency of first sample for each AOI for each facial expression were then extracted. Because the oculometric data were recorded on both eyes individually, the extraction was performed on the eye with the most precise calibration at each experimental block. The accuracy of the children’s responses and their reaction times (i.e., elapsed time until the children pressed the mouse button), were also computed.

Statistical analyses were performed using R software version 4.0.4. The analyses proceeded in four steps. First, response accuracy was analyzed using a GLMER_binomial–logit_ Model (i.e., generalized linear mixed-effects model with binomial logistic regression) built using the “glmer” function (in R lme4 package). The binary variable being right/wrong answer, with facial expression of the avatars and child’s group (i.e., with or without service dog) as the fixed factors, and with the child’s age, sex, and presence/absence of comorbidity, as well as the avatar’s ethnocultural group, age and sex, included as random factors. For the second step, reaction times were analyzed with application of a logit transformation followed by a LMER model (i.e., linear mixed-effect model) using the “lmer” function with the same fixed and random factors as for the response accuracy. Third, for oculometric data, the sample durations within each AOI were analyzed using a GLMER_binomial–logit_ model, with the binary response variable being the time spent in each AOI relatively to the time spent all other AOIs (i.e., proportion) using the “cbind” function (in R base package) within the GLMER model.^2^ The AOI, the facial expression of the avatars and the group were defined as fixed factors, while the children’s age, sex, and presence/absence of comorbidity, as well as the avatar race, age, and sex, were included in the models as random factors. For the fourth step, the latencies of the first sample in each AOI, were analyzed using an LMER model, with the same fixed and random factors as for the durations. At all steps, a type III ANOVA (“Anova” function in R car package) was applied on the model to test for the significance of the fixed factors and their interactions. When relevant, *post hoc* tests were conducted using pairwise comparisons on least-squares means extracted from the models (using “lsmeans” and “cld” functions in R lsmeans and multcomp package), with *p*-values adjusted for multiple comparisons (Tukey method). All tests were two-tailed and the significance threshold was set at *p* ≤ 0.05.

## Results

### Accuracy and Reaction Time

Analyses revealed a significant main effect of facial expression on response (χ^2^ = 15.65, *p* = 0.004), but no main effect of child’s group (χ^2^ = 0.027, *p* = 0.870) nor an interaction (χ^2^ = 5.16, *p* = 0.271) were observed. *Post hoc* tests indicated that children with ASD recognized joy more accurately than fear and sadness (respectively, *z* = 4.75, *p* < 0.001; *z* = 3.67, *p* = 0.002) (Table 2), and that they recognized the neutral expression more accurately than fear (*z* = 3.96, *p* < 0.001).

**TABLE 2:** Response accuracy, reaction time, and mean fixation duration on the six AOI of children with ASD according to their group (i.e., with a service dog, without a service dog, and both groups) and to the displayed facial expression of the avatars.

		Accuracy (±SD in %)	Reaction time (±SD in seconds)	Mean fixation duration for each AOI (± SD in seconds)					
				Left eye	Right eye	Nose	Mouth	Contours	Outside
Anger	With	1.00 ± 0.000	6.464 ± 2.781	1.053 ± 1.193	0.748 ± 0.961	1.458 ± 1.193	2.041 ± 1.260	0.674 ± 1.230	0.274 ± 0.714
	Without	0.923 ± 0.268	7.937 ± 2.876	1.253 ± 1.024	0.815 ± 0.994	1.366 ± 0.949	2.695 ± 1.839	0.910 ± 1.251	0.374 ± 0.635
	Both groups	0.962 ± 0.192	7.162 ± 2.915	1.151 ± 1.115	0.781 ± 0.976	1.413 ± 1.079	2.361 ± 1.601	0.790 ± 1.243	0.323 ± 0.677
Joy	With	0.992 ± 0.091	6.613 ± 2.975	0.479 ± 0.703	0.346 ± 0.596	1.105 ± 1.002	3.388 ± 2.093	0.650 ± 0.991	0.374 ± 0.833
	Without	0.975 ± 0.158	7.380 ± 3.277	0.616 ± 0.857	0.564 ± 0.845	1.060 ± 0.820	3.624 ± 2.573	0.873 ± 1.432	0.509 ± 0.929
	Both groups	0.983 ± 0.129	6.990 ± 3.144	0.548 ± 0.785	0.455 ± 0.738	1.082 ± 0.914	3.506 ± 2.343	0.761 ± 1.234	0.442 ± 0.883
Neutral	With	0.950 ± 0.219	7.705 ± 2.980	0.850 ± 1.079	0.690 ± 0.850	1.455 ± 0.961	3.253 ± 1.923	1.040 ± 1.434	0.325 ± 0.631
	Without	0.924 ± 0.267	7.899 ± 2.981	1.338 ± 1.025	0.838 ± 1.002	1.517 ± 1.126	2.598 ± 1.655	1.182 ± 1.520	0.442 ± 0.713
	Both groups	0.937 ± 0.244	7.800 ± 2.975	1.088 ± 1.078	0.762 ± 0.928	1.486 ± 1.043	2.934 ± 1.824	1.109 ± 1.475	0.382 ± 0.673
Fear	With	0.808 ± 0.400	8.789 ± 2.333	1.055 ± 1.090	0.840 ± 0.992	1.512 ± 1.173	3.761 ± 1.861	0.811 ± 1.218	0.298 ± 0.608
	Without	0.840 ± 0.368	8.366 ± 2.690	1.183 ± 1.246	0.835 ± 0.981	1.238 ± 0.796	3.634 ± 2.058	1.013 ± 1.507	0.424 ± 0.736
	Both groups	0.824 ± 0.381	8.574 ± 2.523	1.117 ± 1.168	0.838 ± 0.984	1.379 ± 1.015	3.699 ± 1.956	0.909 ± 1.367	0.359 ± 0.675
Sadness	With	0.917 ± 0.278	7.961 ± 2.452	0.930 ± 1.095	0.910 ± 0.990	1.415 ± 1.113	3.311 ± 1.731	0.841 ± 1.085	0.258 ± 0.553
	Without	0.849 ± 0.360	8.464 ± 2.611	1.298 ± 1.165	1.091 ± 1.236	1.322 ± 0.919	2.718 ± 1.970	1.134 ± 1.419	0.483 ± 0.784
	Both groups	0.883 ± 0.322	8.202 ± 2.536	1.112 ± 1.143	0.999 ± 1.119	1.369 ± 1.021	3.018 ± 1.872	0.986 ± 1.267	0.369 ± 0.685
All emotion	With	0.933 ± 0.250	7.445 ± 2.855	0.875 ± 1.064	0.708 ± 0.909	1.390 ± 1.099	3.151 ± 1.882	0.804 ± 1.206	0.305 ± 0.673
	Without	0.902 ± 0.298	7.989 ± 2.924	1.135 ± 1.100	0.828 ± 1.030	1.299 ± 0.938	3.056 ± 2.093	1.022 ± 1.429	0.447 ± 0.765
	Both groups	0.918 ± 0.274	7.717 ± 2.890	1.003 ± 1.090	0.767 ± 0.972	1.345 ± 1.023	3.104 ± 1.988	0.911 ± 1.324	0.375 ± 0.723

Analyses on the reaction time did not indicate a main effect of child’s group (χ^2^ = 2.17, *p* = 0.141), but a significant main effect of facial expression was observed (χ^2^ = 36.13, *p* < 0.001) and it interacted significantly with the child’s group factor (χ^2^ = 15.93, *p* = 0.003). We observed that children without a service dog recognized joy faster compared to fear (*t* = 4.96, *p* < 0.001), sadness (*t* = 4.39, *p* < 0.001), and neutral (*t* = 3.19, *p* = 0.01). They also recognized anger faster compared to fear and sadness (respectively, *t* = 3.83, *p* = 0.001; *t* = 3.27, *p* = 0.01) (Table 2). Children with a service dog recognized anger and joy faster than all other emotions (respectively, 5.76 ≤ *t* ≥ 9.16, 4.76 ≤ *t* ≥ 8.19, all *p* < 0.001), but were slower to recognize fear compared to all other emotions (3.22 ≤ *t* ≥ 9.16, all *p* ≤ 0.01). No significant difference between groups was observed for each expression when conducting *post hoc* comparisons (all *p* > 0.05).

### Gaze Duration

Concerning the duration of gaze samples within each AOI, the GLMER highlighted the presence of a significant main effect of the AOI (χ^2^ = 218.71, *p* < 0.001), as well as a significant interaction between facial expression and AOI factors (χ^2^ = 66.84, *p* < 0.001) and between child’s group and AOI factors (χ^2^ = 14.37, *p* = 0.013). It also highlighted a triple interaction between the AOI, the child’s group and the facial expression factors (χ^2^ = 36.16, *p* = 0.015).

*Post hoc* tests on the interaction between facial expression and AOI revealed first, that the mouth was the most visually explored area, followed by the nose area, compared to all other AOI across all facial expressions (respectively, 2.86 ≤ *z* ≥ 22.64, 5.11 ≤ *z* ≥ 16.52, all *p* ≤ 0.05). The outside area was the least visually explored compared to all other AOI across all expressions (3.86 ≤ *z* ≥ 21.10, all *p* ≤ 0.002), except joy as it did not differ from the left and right eye areas (respectively, *z* = 2.04, *z* = 1.70, both *p* > 0.05). The right eye area was less explored than the contours area (3.94 ≤ *z* ≥ 8.97, all *p* ≤ 0.001) across all expressions, as well as than the left eye area (3.73 ≤ *z* ≥ 6.07, all *p* ≤ 0.003) across all expressions, except sadness (*z* = 2.42, all *p* > 0.05) (Table 2). For joy, contours were more explored compared to left eye (respectively, *z* = 5.27, *p* < 0.001), while no difference was observed between both areas for the other expressions (all *p* > 0.05).

*Post hoc* on the interaction between AOI and child’s group showed that children without a service dog visually explored the left eye area significantly longer (*z* = 2.70, *p* = 0.007) as well as the outside area (*z* = 4.06, *p* < 0.001), compared to children with a service dog (Table 2).

**FIGURE 2:**
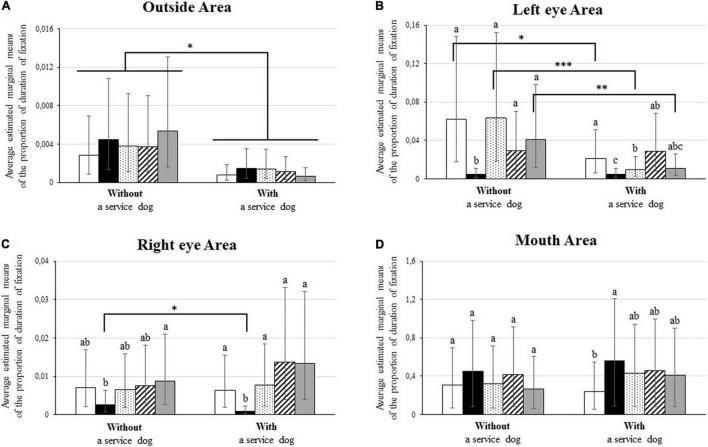
Mean proportion of fixation of children with ASD according to children’s group and to the displayed facial expression for: **(A)** the outside area, **(B)** the left eye area, **(C)** the right eye area, and **(D)** the mouth area. Facial expressions are represented in color code: anger in white, joy in black, neutral in dotted pattern, fear in striped pattern, and sadness in gray. Lower case-letters and stars refer significant differences. Lower-case letters refer significant results from comparisons between expressions within in each group. Data (i.e., facial expressions) referring different letters within the graph differed significantly at *p* ≤ 0.05, while data that share a common letter did not. Stars refer to results from comparisons between groups within each facial expression. *p ≤ 0.05, ^**^p ≤ 0.01, ^***^p ≤ 0.001.

Inspection of the triple interaction highlighted differences between children with ASD with and without a service dog in the time spent exploring the different AOIs according to the displayed facial expressions (see Figure 2). Indeed, compared to children with a service dog, children without a service dog spent significantly more time visually exploring (1) the right eye area for joy (*z* = 2.06, *p* = 0.039), (2) the left eye area for anger, neutral and sadness expressions (respectively, *z* = 2.28, *p* = 0.022; *z* = 3.97, *p* < 0.001; *z* = 2.82, *p* = 0.005), and (3) the outside of the face for all facial expressions (2.00 ≤ *z* ≥ 4.28, all *p* ≤ 0.05). Analyses also revealed that differences in the exploration of the AOIs according to facial expression were not the same in the two groups. First, children with a service dog explored the left eye area more for fear and anger than for joy (respectively, *z* = 4.63, *z* = 3.87; both *p* ≤ 0.001), and less for the neutral expression than for fear (*z* = 2.76; *p* = 0.05), whereas children without a service dog explored the left eye area less for joy than for all other expressions (4.62 ≤ *z* ≥ 6.64, all *p* < 0.001). Second, children with a service dog explored the right eye less for joy than all other expressions (4.69 ≤ *z* ≥ 6.61, all *p* < 0.001), whereas children without a service dog explored it less for joy than sadness (*z* = 2.96; *p* = 0.03). Third, children with a service dog explored the mouth area more for joy than for anger (*z* = 3.61; *p* = 0.003), whereas no difference was observed for children without a service dog (all *p* > 0.05).

### Gaze Sample Latency

Concerning results on the latency of the first gaze sample, these results highlighted the presence of a significant main effect of the AOI factor (χ^2^ = 231.88, *p* < 0.001), but no significant effect of the facial expression factor (χ^2^ = 9.25, *p* > 0.05). They also showed an interaction between the AOI and facial expression factors (χ^2^ = 51.64, *p* < 0.001), and between the AOI and group factors (χ^2^ = 12.04, *p* = 0.03). The triple interaction between the AOI, the facial expression and the group factors was not significant (χ^2^ = 25.47, *p* > 0.05).

*Post hoc* on the interaction between AOI and facial expression revealed that children with ASD’s latency of first fixation to the different AOI varied according to the facial expression displayed by the avatar. Those analyses indicated that children with ASD gazed: (1) faster at the nose area (*M* = 0.58 ± 1.67 s, 6.21 ≤ *t* ≥ 19.79, all *p* < 0.001) and the mouth area (*M* = 1.02 ± 1.51 s, 4.72 ≤ *t* ≥ 19.22, all *p* < 0.001), across all expressions; (2) slower at the outside of the face area (*M* = 5.83 ± 4.40 s, 5.82 ≤ *t* ≥ 18.37, all *p* < 0.001) and the right eye area (*M* = 5.46 ± 4.26 s, 3.45 ≤ *t* ≥ 15.86, all *p* ≤ 0.007), across all expressions, except for joy for which the right eye area was the slowest (*M* = 6.60 ± 4.29 s; 3.40 ≤ *t* ≥ 19.79, all *p* ≤ 0.009); (3) they also gazed faster at the contour area compared to the left eye for joy (*M* = 3.24 ± 3.69 s vs 5.31 ± 4.53 s, *t* = 6.77, *p* < 0.001), fear (*M* = 2.44 ± 2.94 s vs 3.88 ± 3.87 s, *t* = 4.72, *p* < 0.001) and sadness (*M* = 2.73 ± 3.22 s vs 4.034 ± 4.13 s, *t* = 4.24, *p* < 0.001), with a similar tendency for the neutral expression (*M* = 2.77 ± 3.40 s vs 3.65 ± 4.05 s, *t* = 2.79, *p* = 0.06).

Analyses on the interaction between the AOI and the child’s group revealed that, compared to children with a service dog, children without a service dog gazed faster at the left eye and outside areas (respectively, *M* = 3.40 ± 3.91 s vs 4.75 ± 4.16 s, *t* = 2.71, *p* = 0.01; *M* = 2.60 ± 3.26 s vs 3.03 ± 3.33 s, *t* = 2.68, *p* = 0.01).

## Discussion

Our aim was to investigate if the daily presence of a service dog had an impact on the facial expression processing skills of children with ASD. Two groups of 15 children with ASD, one with a service dog and the other without, were compared using a computerized facial expression recognition task. The two groups did not differ significantly in their accuracy and reaction time. However, results suggest that both groups differed in their general attentional allocation toward the different facial features, as well as in their variation of scanning strategies according to the displayed facial expression.

### Speed and Accuracy of Facial Expression Recognition

In line with past researches (Smith et al., 2010; Lozier et al., 2014; Evers et al., 2015; Wingenbach et al., 2017), we globally observed that children with ASD recognized joy more efficiently (i.e., higher accuracy and shorter reaction times) compared to negative expressions, notably fear and sadness. Although direct comparisons between both groups did not turn out to be significant, potentially because of lack of power due to our small sample size, results highlight that children with ASD without a service dog recognized joy faster than fear, sadness, and neutral expression, whereas children with a service dog recognized joy and anger faster than all other expressions. Children with a service dog could thus be slightly quicker to recognize anger than children without a service dog.

Some hypotheses may explain the absence of significant difference between children with and without a service dog on both their response accuracy and their reaction time. First, we may interpret the absence of significant difference between both groups as indicative of an absence of impact of the service dog on these measures. Second, it may be explained by a ceiling effect which may have reduced differences between the two groups. Indeed, it is noteworthy that in the present study children with ASD had high accuracy rates along with relatively high reaction times (mean reaction time across expressions of 7.7 s). This could indicate that children privileged a more cautious strategy: they took longer to answer in order to look more at the avatar and optimize their accuracy. Additional elements may have contributed to this ceiling effect such as the task participants had to perform along with the procedure and type of stimuli used (i.e., dynamic prototypical facial expression displayed by avatars, with long exposure, morphed expression with maximal intensity expressivity), which may have facilitated the recognition of facial expressions for children with ASD in the present study. Previous studies have highlighted the impact of those type elements [i.e., type of the task, nature of the stimuli (e.g., static vs dynamic), intensity of expressions] on facial expression processing in individuals with ASD (Speer et al., 2007; Guillon et al., 2014; Mouga et al., 2021; Nagy et al., 2021). Future studies on similar issues should carefully consider those elements in order to produce enough variation to optimize the evaluation of an effect, for example by increasing the presentation speed (Matsumoto and Hwang, 2011).

### Visual Exploration of Facial Features

Concerning the exploration of facial features, children with ASD in both groups gazed faster and explored the mouth and nose areas longer, while they gazed slower and explored the outside area less compared to all other facial features. Previous researches on children with ASD have reported a deficit in the exploration of faces compared to the rest of the visual scene (Klin et al., 2003; Riby and Hancock, 2008; Vacas et al., 2021). Results from the present study do not necessarily contradict those findings, because the outside area was blank and did not contain items (e.g., objects, furniture, and decor) that may potentially attract children’s attention. Furthermore, the present results are in line with studies reporting a deficit in the exploration of the eyes area (Speer et al., 2007; Corden et al., 2008; Chita-Tegmark, 2016; Black et al., 2017) and an increased focus on the mouth area (Joseph and Tanaka, 2003; Dalton et al., 2005; Spezio et al., 2007; Bird et al., 2011). It is however important to highlight that this imbalance in the exploration of the eyes and mouth in individuals with ASD is debated (Guillon et al., 2014; Chita-Tegmark, 2016).

Results also highlighted that both groups differed in their general scanning of faces. Indeed, children without a service dog spent more time exploring and gazed faster at the left eye and the outside area (i.e., rest of the visual scene) compared to the group with a service dog. Thus, even though children without a service dog explored the eyes area more, it seems that they also pay more attention to an area that is not relevant to facial expression processing (i.e., the outside area).

### Variation of Scanning According to the Displayed Facial Expression

As expected based on the previous studies (de Wit et al., 2008; Åsberg et al., 2017), both groups of children with ASD displayed variations in their scanning strategies (i.e., visual exploration of the different facial features) according to the displayed facial expression. Indeed, both groups explored the eye areas less when the face displayed joy compared to when a negative expression was displayed. However, only the group with a service dog displayed modulation according to the displayed expression for the mouth area: they spent more time exploring this area for joy compared to anger. Furthermore, different studies in eye-tracking on NT individuals have reported a dichotomy in the exploration of the eye and mouth areas between anger and joy (Calvo and Nummenmaa, 2008; Eisenbarth and Alpers, 2011; Schurgin et al., 2014; Calvo et al., 2018). In the present study, only the group with a service dog exhibited a similar dichotomy: they explored the eye areas more for anger than for joy, and explored the mouth area more for joy than for anger. Thus, even though both groups adapted their scanning strategies according to the displayed expression, it seems that children with a service dog displayed scanning strategies that are more differentiated according to the expression, and which looks more similar to what has been observed in NT individuals. Results from this pilot study could thus be indicative that having a service dog and benefiting from its daily presence during multiple years may have an impact on the facial expression visual processing strategies of children with ASD.

### Potential Source of This Effect of Service Dog on Children With Autism Spectrum Disorder’s Scanning Strategies

From a theoretical point of view, it has been suggested that the benefit of animals on the psychosocial development of children with ASD may arise from a generalization process. Meaning that children with ASD will extend and apply in their interaction with humans the skills they developed and trained while interacting with the animal (Filiâtre et al., 1986; Grandgeorge et al., 2012, 2020). In our case, such a generalization process could notably be promoted by the attraction toward animals and their face observed in children with ASD (Martin and Farnum, 2002; Prothmann et al., 2009; Grandgeorge et al., 2012; Carlisle, 2015; Valiyamattam et al., 2020). Similar brain areas are involved for the processing of human and animal faces and the recruitment of those areas is not altered for the processing of animal faces in individuals with ASD (in NT: Blonder et al., 2004; in ASD: Whyte et al., 2016). Previous studies have also shown that children with ASD do not have difficulties in orienting their attention toward animal faces (Dollion et al., 2021) and exploring their core features, notably the eyes (Muszkat et al., 2015; Grandgeorge et al., 2016; Valiyamattam et al., 2020). Additionally, contrarily to human faces, children with ASD do not show a deficit in the recognition of facial expression displayed by dog faces compare to NT children (Davidson et al., 2019). Having a service dog and processing its face and facial expression could thus provide children with ASD with daily opportunities that will recruit and train circuits, strategies and cognitive skills that are involved in facial expression processing; and that they may generalize to human faces afterward. Thus, the fact that children with ASD with a service dog spent less time on areas that are not relevant to the processing of facial expression (i.e., the outside area) compared to children without a service dog, could thus be explained by a generalization of the visual strategies they may have exerted and trained with their service dog’s face for multiple years.

However, based on this assumption of a generalization process taking place, we would expect that children with a service dog would also gaze more at the eye area of human faces, since previous studies have shown that children with ASD explore the eyes of animal faces more (Grandgeorge et al., 2016; Valiyamattam et al., 2020). On the contrary, results from the present study indicate that the group with a service dog took longer to and gazed less at the left eye compared to the group without a service dog, and that they displayed a differential exploration of the mouth area according to the displayed expression; while children without a service dog did not. Yet, contrarily to human facial expression, dog facial expression relies on the activation of facial muscles located on the lower part of the face, and notably around the mouth area (Caeiro et al., 2017; Waller et al., 2020). The recognition of dog’s facial expression would thus rely more on the visual exploration of the mouth area. Consequently, we may hypothesis that by benefiting from the daily presence of their service dog and processing their facial expressions for multiple years, children with ASD may have developed strategies engaging greater attention, and differential scanning, on the mouth area, with their service dog, that they may then generalize and engage when processing human facial expression.

Additionally, the observed differences in the scanning of the mouth area between both groups could explain why children with ASD with a service dog seemed to be slightly quicker to recognize anger than children with ASD without a service dog in the present study. Efficient distinction and recognition of facial expressions involve the visual exploration of expression-distinctive specific facial features, and thus variation in scanning strategies according to the displayed emotion with an increase focus on the diagnostic area(s) (Calder et al., 2000; Calvo and Nummenmaa, 2008; Calvo et al., 2018; Beaudry et al., 2014; Wells et al., 2016; Blais et al., 2017). It has been hypothesized that enhanced selective visual attention on diagnostic regions of the respective expressions enhances/facilitates facial expression recognition (Schurgin et al., 2014; Calvo et al., 2018). In the present study, only children with ASD from the with a service dog group displayed differences in their exploration of the mouth according to the displayed expression (i.e., joy vs anger) and only this group displayed a dichotomy in their exploration of the eye and mouth areas between anger and joy. We may thus hypothesize that children with ASD with a service dog having more distinctive strategies for anger and joy, it may have improved the distinctiveness of those expressions for those children, which in turn promoted/facilitated their recognition for them.

More generally, results from this pilot study seem to indicate that having a service dog and benefiting from its daily presence may promote the development of specific visual exploration strategies for the processing of human faces.

## Strengths, Limitations, and Perspectives

The present pilot study is the first to investigate if having a service dog within the daily life of children with ASD have an influence on their facial expression recognition skills. Relying on objective measurements through a computerized facial expression recognition task coupled with an eye-tracker, results of this study highlights that having a service dog may have an effect on the visual strategies for the processing of facial expression in children with ASD. Even so both groups did not differ in their accuracy and reaction time to recognize facial expression, we observed that children without a service dog notably spent more time exploring a non-relevant area for facial expression processing (i.e., outside the face) compared to children with a service dog. Furthermore, we observed that only children with a service dog displayed variation in their scanning of the mouth area according to the displayed emotion, as well as a dichotomy between joy and anger in their exploration of the eye and mouth areas. It thus seems that children with ASD with a service dog displayed more suited and more differentiated visual scanning strategies when processing facial expressions. More generally, the present preliminary results seem to indicate that the presence of a service dog, and maybe pets in general, might be a parameter to take into consideration for future investigation of face processing in children with ASD. Also, and more importantly, they seem to indicate that being the beneficiary of a service dog may have an impact on how children with ASD decode facial expression. The impacts of animals on the communication and interaction skills of children with ASD may thus also extend to facial expressions processing skills.

However, a number of limitations have to be addressed. First, it is important to nuance the present results since they were collected on a relatively small number of individuals. Further confirmation of these preliminary results through studies on a larger sample to confirm these results, along with further investigation on the mechanisms associated with these effects on facial expression processing strategies in children with ASD, are thus necessary.

Second, all general information relative to children with ASD and their family were gathered through access to children’s full record at the Mira Foundation. However, these records did not include current information relative to the actual presence of pets within the family household or recent assessment of ASD severity. Previous studies have demonstrated that difficulties in facial expression recognition and alteration in face visual exploration are positively associated with ASD severity (e.g., Evers et al., 2015; Åsberg et al., 2017). It could thus be of interest for future studies to explore if the impact of service dog on the processing of facial expressions varies according to ASD severity. Additionally, future studies should compare if similar results are observed between dogs and other species of pets, as well as between a service dog and pet dogs, because it will allow the possibility of investigating if these effects are specific to service dogs.

Although we checked for the presence/absence of comorbidity based on consultation of participants’ full medical record, it could have been of interest to test for mood disorders and other psychological disorders (notably anxiety and depression) in our participants using standardized scales, since those type of disorders may have an impact on facial expression processing (e.g., Leppänen, 2006; Demenescu et al., 2010; Collin et al., 2013). The same goes for children with ASD’s current medication status (e.g., methylphenidate and benzodiazepine), which was not collected by the time of experimentation and which may also affect the results (e.g., Conzelmann et al., 2011). Collecting both information would have allowed to test and control – in our model – for the specific effects associated with those disorders and medication.

Furthermore, we did not have access to information relative to the types of therapeutic interventions children may have had access to in the past or by the time of experimentation, as well as their involvement in activity groups aiming at promoting communication, social skills and the understanding of facial expressions. Similarly, we did not collect information relative to families’ socioeconomic status, which affect families’ investment in and resources for rehabilitation activities for their child.

Finally, the present study was cross-sectional. It would be of interest to replicate this study in the context of a longitudinal study. Such design would allow exploration and comparison of facial expression processing skills and visual exploration strategies of children with ASD prior to and after service dog attribution, as well as the long-term effect of such intervention.

## Conclusion

To conclude, our study showed for the first time that having a service dog and interacting with it on a daily basis may promote the development of specific visual exploration strategies for the processing of human faces, highlighting the real interest that animals could have in the daily life of children and adolescents with ASD.

## Data Availability Statement

The raw data supporting the conclusions of this article will be made available by contacting the corresponding author. Requests to access these datasets should be directed to ND, nicolas.dollion@univ-rennes1.fr.

## Ethics Statement

The studies involving human participants were reviewed and approved by the Research Ethics Committee in Education and Psychology, University of Montréal. Written informed consent to participate in this study was provided by the participants’ legal guardian/next of kin.

## Author Contributions

ND, PP, and MG developed the research project and contributed to the statistical analysis. ND, PP, DS-A, AH, and NMF designed and built the experiment. NC, NF, and ND organized the recruitment of our study subjects. ND performed the experiments. ND and AH performed the data extraction and analyses. All authors participated in the writing of the manuscript and proofread the final version.

## Conflict of Interest

The authors declare that the research was conducted in the absence of any commercial or financial relationships that could be construed as a potential conflict of interest.

## Publisher’s Note

All claims expressed in this article are solely those of the authors and do not necessarily represent those of their affiliated organizations, or those of the publisher, the editors and the reviewers. Any product that may be evaluated in this article, or claim that may be made by its manufacturer, is not guaranteed or endorsed by the publisher.

## Footnotes

1 Service dogs for children with ASD are a specific type of service dogs that have been specifically selected and that have received specific training in order to accompany/assist children with ASD in their daily-life and across various activities with the aim to increase/improve their functional abilities (Harris and Sholtis, 2016; Guerin, 2020).

2 Here, each msec spent gazing at the screen for each trial was considered as a single measurement point. Thus, each measurement point could take either the value 1 (when the gaze was detected within the AOI) or the value 0 (when the gaze was detected within any of the others AOIs). R cbind function was used to build, and insert within the model, a two columns matrix including the number of successes (number of measurement points with gaze detected within the AOI) and failures (number of measurement points with gaze detected within the other AOIs). This way, gaze measurement becomes a binary variable since it corresponds to the number of msec gazing in the AOI relatively to the total number of msec gazing in all AOIs. This statistical methodology was used due to the strictly bounded nature of the response variable (i.e., the relative frequency of gazing in the AOI relatively to all other AOIs being ≥0 and ≤1).

## Supplementary Material

Supplementary File 1 Example of one of the videos used as stimuli in the experiment. Video of a female adult avatar with African ethnocultural characteristics displaying joy.
